# Robust Fixed-Time H∞ Trajectory Tracking Control for Marine Surface Vessels Based on a Self-Structuring Neural Network

**DOI:** 10.1155/2022/6515773

**Published:** 2022-07-07

**Authors:** Xuehong Tian, Zhicheng Wang, Jianbin Yuan, Haitao Liu

**Affiliations:** ^1^School of Mechanical and Power Engineering, Guangdong Ocean University, Zhanjiang 524088, China; ^2^Shenzhen Institute of Guangdong Ocean University, Shenzhen 518120, China

## Abstract

In this study, a robust fixed-time H∞ trajectory tracking controller for marine surface vessels (MSVs) is proposed based on self-structuring neural network (SSNN). First, a fixed-time H_∞_ Lyapunov stability theorem is proposed to guarantee that the MSV closed-loop system is fixed-time stable (FTS) and the *L*_2_ gain is less than or equal to *γ*. This shows high accuracy and strong robustness to the approximation errors. Second, the SSNN is designed to compensate for the model uncertainties of the MSV system, marine environment disturbances, and lumped disturbances term constituted by the actuator faults (AFs). The SSNN can adjust the network structure in real time through elimination rules and split rules. This reduces the computational burden while ensuring the control performance. It is proven by Lyapunov stability that all signals in the MSV system are stable and bounded within a predetermined time. Finally, theoretical analysis and numerical simulation verify the feasibility and effectiveness of the control scheme.

## 1. Introduction

With the rise of marine activities such as sea rescue, military reconnaissance, and environmental monitoring, surface unmanned vessels play an important role in the field of marine engineering. Among them, trajectory tracking is a popular control problem [1–7]. However, with wind, waves, and currents having a significant impact on the speed and maneuverability of vessels, achieving safety, accuracy, and stability is a significant challenge for control system design. Exploring better control schemes to achieve fast stability and strong robustness of MSV systems requires further research.

Finite-time stabilization has a faster response speed than asymptotic stabilization, which ensures that the tracking error is stabilized within a small range near zero within a limited time, so it has strong robustness. Relying on this advantage, a robust control scheme based on a finite-time disturbance observer was designed [8]. A control scheme combining a finite-time disturbance observer and sliding mode technique effectively suppresses disturbances [9]. A new finite time-expanded observer was proposed to facilitate the observation of lumped disturbances caused by uncertainties and external disturbances [10]. Considering the uncertainty of the model and the disturbance of the marine environment, a semiglobal finite-time stable control strategy was designed by combining the disturbance observer and adaptive neural network (NN) technology [11]. However, it should be pointed out that the settling time of the finite-time control scheme depends on the initial state of the controlled object; that is, when the initial position is far from the target position, the system takes longer to reach stability. Affected by this, the concept of fixed-time stability was proposed [12]; its setting time is related to the designed controller parameters and is not influenced by the initial state of the MSV.

At present, researchers have proposed some control schemes based on fixed-time stability, such as a combined method of fixed-time extended state observer (FTEXSO) and increased power integration [13], homogeneous technology [14], and sliding mode control based on FTEXSO [15, 16]. However, the nonsmooth control signal provided by the timing controller based on sliding mode surface control causes inherent chattering, which is harmful to the mechanical structure and electronic equipment of the MSV system. Controllers based on homogeneous technology cannot guarantee globally fixed-time stability. These control schemes have strong robustness to unknown disturbances and uncertainties, but they do not discuss the relationship between control parameters and robustness. This relationship is explained in the H_∞_ control [17, 18]. The robustness of H_∞_ control makes the system internally stable, but more important, it can control the output of the controlled object to meet a prespecified upper limit of gain by suppressing external disturbances [19]. Due to its good characteristics, the H_∞_ controller has been widely studied in linear systems, switching systems, and extracorporeal blood circulation systems [20] and applied to robotic manipulator systems [21–23]. However, there have been few attempts to introduce H_∞_ control technology into MSV systems.

In the complex ocean environment, the uncertainty of the MSV system and environmental disturbances are indispensable. For complex nonlinear systems, an evolutionary bat algorithm (EBA) controller based on artificial intelligence was proposed to stabilize the fuzzy system through affine transformation and parameter linear matrix inequality [24]. Recently, the use of NN to compensate for unknown nonlinear functions of the system has become more promising. Radial basis function neural networks (RBFNNs) have good fitting performance and are one of the most common neural networks [25, 26]. As we all know, the fitting ability and calculation amount of NN are closely related to the network structure, and the complexity of the nonlinear function often determines the complexity of the network structure. A simple network structure may lead to poor fitting ability and cannot meet the requirements, while an overly complex network structure causes a computational burden. How to match the optimal network structure according to the complexity of nonlinear functions is a problem worth exploring [27–29]. In addition, it must be considered in the controller design that the actuator may fail, resulting in the degradation or even failure of the control performance [15].

Based on the above discussion, we improve the trajectory tracking accuracy of MSV and the stability of the control system and help the development of marine activities. The purpose of this study is to provide a fixed-time robust control strategy for the trajectory tracking control system of an MSV with marine environment disturbance, vessel model uncertainty, and AF. An SSNN is used to approximate the lumped disturbances caused by external disturbances, AF, and uncertainty and can optimize the network structure online, which is conducive to improving the fitting accuracy and avoiding the computational burden. The stability of the control system is improved by suppressing the interference through H_∞_ control. Finally, based on the fixed-time stability theory, it is proven that the tracking error can converge in a fixed-time when all closed-loop signals are bounded. References [30, 31] are typical MSV control algorithms based on neural network control. Compared with [30], the proposed control scheme can greatly improve the system response speed. Compared with [31], the upper bound of the convergence time of the proposed control scheme does not depend on the initial state of the system. The main contributions of this paper are listed as follows:

1. A fixed-time H_∞_ robust control scheme based on an SSNN is proposed for the trajectory tracking problem of MSVs. The scheme can ensure that the MSV accurately tracks the desired trajectory, thus achieving bounded convergence of the tracking error within a fixed-time and making the convergence time independent of the initial state. To improve the robustness of the control system, a fixed-time H_∞_ is proposed to study the control problem. The controller can provide disturbance attenuation in the sense of *L*_2_ gain without solving complex Hamilton-Jacobian equations or inequalities or Riccati equations.
2. Based on the traditional RBFNN algorithm, the network structure is optimized online by establishing a splitting rule and eliminate rule, and then an SSNN is proposed to approximate the nonlinear term of the system. Compared with RBFNN, SSNN can effectively reduce the amount of calculation and save network resources under the premise of ensuring good fitting accuracy.
3. The influence of the AF on the controller is analyzed, and the solution is given.

The rest of this article is organized as follows.  Section 2 presents the preparation of the MSV problem formulation.  Section 3 introduces the design idea of a robust fixed-time H_∞_ control scheme based on the SSNN and a stability analysis of the control system. In Section 4, the effect of actuator faults on the controller is analyzed, and the proposed SSNN is verified by comparison.  Section 5 summarizes the research and suggests future work.

## 2. Preliminaries and Problem Formulations

### 2.1. Preliminaries

*Notation*. Defining |*x*|=[|*x*_1_|, |*x*_2_|,…,|*x*_*n*_|]^*T*^, *x* ∈ *R*^*n*^, |*·*| represents the absolute value of a scalar or vector component. ‖*·*‖ represents the vector Euclidean 2-norm. Denoting *sig*^*a*^(*x*)=*sign*(*x*)|*x*|^*a*^, diag{*·*} indicates a diagonal matrix.

*Neural Networks (NNs)*. Suppose *f*_*NN*_(*χ*) : *U*_*f*_^*n*^⟶*R* is an unknown nonlinear function that can be estimated by NN on a compact Ω ∈ *U*_*f*_^*n*^ as follows:

(1) $$\begin{array}{c} f_{NN}\left(\chi \right)=W^{*T}\Phi \left(\chi \right)+\varepsilon \left(\chi \right), \end{array}$$

where *ε* ∈ *R*^3^ is the fitting error, *W*^*∗*^ ∈ *R*^*m*^ is the ideal weight, and

(2) $$\begin{array}{c} W^{*}=\arg\underset{\hat{W}}{\min}\left\{\underset{\chi \in \Omega }{\sup}\left|f_{NN}\left(\chi \right)-{\hat{W}}^{T}\Phi \left(\chi \right)\right|\right\}, \end{array}$$

where $\hat{W}$ is the weight estimate value of the NN, Φ(*χ*)=[*h*_1_(*χ*),…,*h*_*m*_(*χ*)]^*T*^ : Ω⟶*R*^*m*^ represents the activation value of the NN, *χ* represents the input of the NN, and the activation function is as follows:

(3) $$\begin{array}{c} h_{i}\left(\chi \right)=\text{exp}\left(-\frac{{\left\|\chi -c_{i}\right\|}^{2}}{j_{i}^{2}}\right),\;i=1,\ldots ,m, \end{array}$$

where *c*_*i*_ ∈ *U*_*f*_^*n*^ and *j*_*i*_ ∈ *R* indicate the center coordinates and width of the *i*th neuron, respectively.

*Actuator Faults (AF)*. The actuator faults of the controller can be achieved by the following rule:

(4) $$\begin{array}{c} \tau_{ni}=\tau_{i}+b_{i}\left(t-t_{0i}\right)\left(\left(e_{ii}-1\right)\tau_{i}+{\bar{\tau }}_{ni}\right), \end{array}$$

where *τ*_*ni*_ represents the actual control input, and *τ*_*i*_ represents the input required by the designed controller. *e*_*ii*_ represents the health condition of the *i*th actuator and satisfies 0 ≤ *e*_*ii*_ ≤ 1. ${\bar{\tau }}_{ni}$ indicates other unknown faults. The time-varying distribution function *b*_*i*_(*t* − *t*_0*i*_) is defined as follows:

(5) $$\begin{array}{c} b_{i}\left(t-t_{0i}\right)=\left\{\begin{array}{cc} 0, & t<t_{0i},\\ 1-e^{-a_{i}\left(t-t_{0i}\right)}, & t\geq t_{0i}. \end{array}\right. \end{array}$$

**Assumption 1 .**

1. The desired trajectory *n*_*d*_=[*x*_*d*_, *y*_*d*_, *ψ*_*d*_]^*T*^ is differentiable, and *n*_*d*_, ${\dot{n}}_{d}$, and ${\ddot{n}}_{d}$ are bounded [13].
2. The disturbance *d*(*t*) in (23) is bounded, and $\left\|d\left(t\right)\right\|\leq \bar{d}$, where $\bar{d}$ are unknown positive constants [32].

### 2.2. Definitions and Lemmas

**Definition 1 .**

(see [12]). The system is given as follows:

(6) $$\begin{array}{c} \dot{x}=f\left(x\left(t\right)\right),f\left(0\right)=0,x\in R^{n}, \end{array}$$

where $f:\overset{⌢}{U}⟶R^{n}$ is continuous on an open neighborhood *U*_0_ of the origin. The equilibrium *x*=0 of system (6) is (locally) FTS if (i) it is Lyapunov stable and finite-time convergent in a neighborhood ${\overset{⌢}{U}}_{0}\in \overset{⌢}{U}$ of the origin; (ii) it is fixed-time convergent in $\overset{⌢}{U}$, that is, every solution *x*(*t*, *x*_0_) of system (6) satisfies $x\left(t,x_{0}\right)\in {\overset{⌢}{U}}_{0}/\left\{0\right\}$ for *t* ∈ [*t*_0_, *T*), where *T* represents the settling time and satisfies

(7) $$\begin{array}{c} \underset{t⟶T}{\lim}x\left(t,x_{0}\right)=0. \end{array}$$

If $\overset{⌢}{U}=R^{n}$ and *t* ≥ *T*, then the origin of system (6) is globally FTS.

The following definition is proposed here:

**Definition 2 .**

Analyze a closed-loop system with the following form:

(8) $$\begin{array}{c} \left\{\begin{array}{c} \dot{x}=f\left(x\right)+\omega \left(x\right)+g\left(x\right)u\\ z=\hbar \left(x\right) \end{array}\right., \end{array}$$

where *x* ∈ *R*^*n*^ is the state variable of the system, *u* ∈ *R*^*m*^ is the system input, *ω* ∈ *R*^*r*^ represents indeterminate disturbance, and *z* ∈ *R*^*r*^ is a vector of performance metrics. As a globally fixed-time H_∞_ controller, the following compensators exist:

(9) $$\begin{array}{c} u=f_{u}\left(x,t\right), \end{array}$$

and setting a positive arithmetic number *γ* > 0, system (8) has *L*_2_ gain less than *γ* if for all *ω* ∈ *L*_2_[*t*_0_, *t*_1_] satisfies

(10) $$\begin{array}{c} \int_{t_{0}}^{t_{1}}{\left\|z\left(t\right)\right\|}^{2}dt\leq \gamma^{2}\int_{t_{0}}^{t_{1}}{\left\|\omega \left(t\right)\right\|}^{2}dt, \end{array}$$

where *z*(*t*) is the system output with initial condition *x*(*t*_0_)=0.

**Remark 1 .**

If there is a control input *u*(*t*) in the range [*t*_0_, *t*_1_] such that system (8) has a fixed-time H_∞_ performance, then *u*=*f*_*u*_(*x*, *t*) can be called the fixed-time robust H_∞_ controller of system (8) so that the *L*_2_ gain equal to or less than *γ*, and the robustness performance specification can be expected by choosing an appropriate value of *γ*.

**Lemma 1 (see [12]).**

Suppose *V*(*x*) is a positive Lyapunov function and satisfies $\dot{V}\left(x\right)\leq -\kappa_{1}V{\left(x\right)}^{\alpha }-\kappa_{2}V{\left(x\right)}^{\beta }$; then, system (6) is an FTS, where *κ*_1_ > 0, *κ*_2_ > 0 and 0 < *α* < 1, *β* > 1. The settling time satisfies *T* ≤ *T*_*MAX*_=1/*κ*_1_(1 − *α*)+1/*κ*_2_(*β* − 1).

**Lemma 2 (see [33]).**

Suppose *V*(*x*) is a positive Lyapunov function and satisfies $\dot{V}\left(x\right)\leq -\kappa_{1}V{\left(x\right)}^{\alpha }-\kappa_{2}V{\left(x\right)}^{\beta }+C$; then, system (6) is practically fixed-time stable (PFTS), where *κ*_1_ > 0, *κ*_2_ > 0, 0 < *C* < *∞* and 0 < *α* < 1, *β* > 1. The settling time satisfies

(11) $$\begin{array}{c} T\leq T_{\text{MAX}}≔\frac{1}{\kappa_{1}g\left(1-\alpha \right)}+\frac{1}{\kappa_{2}g\left(\beta -1\right)}, \end{array}$$

where 0 < *g* < 1, and the set of residuals of system (6) is as follows:

(12) $$\begin{array}{c} x\in \left\{V\left(x\right)\leq \min\left\{{\left(\frac{C}{\left(1-g\right)\kappa_{1}}\right)}^{1/\alpha },{\left(\frac{C}{\left(1-g\right)\kappa_{2}}\right)}^{1/\beta }\right\}\right\}. \end{array}$$

**Lemma 3 (see [17]).**

If 0 < *ς*_1_ < 1 and 0 < *ς*_2_ < 2, for ∀*x*_*i*_ ∈ *R*, *i*=1,…, *n*, then

(13) $$\begin{array}{c} {\left(\left|x_{1}\right|+\cdots +\left|x_{n}\right|\right)}^{\varsigma_{1}}\leq {\left|x_{1}\right|}^{\varsigma_{1}}+\cdots +{\left|x_{n}\right|}^{\varsigma_{1}}, \end{array}$$

(14) $$\begin{array}{c} {\left({\left|x_{1}\right|}^{2}+\cdots +{\left|x_{n}\right|}^{2}\right)}^{\varsigma_{2}}\leq {\left({\left|x_{1}\right|}^{\varsigma_{2}}+\cdots +{\left|x_{n}\right|}^{\varsigma_{2}}\right)}^{2}. \end{array}$$

**Lemma 4 .**

Assume $\hat{\varpi }=-\tilde{\varpi }+\varpi^{*}$, *l* > 1/2; then, the following inequality is true:

(15) $$\begin{array}{c} \tilde{\varpi }\hat{\varpi }=-{\tilde{\varpi }}^{2}+\tilde{\varpi }\varpi^{*}\leq \left(-{\tilde{\varpi }}^{2}+\frac{1}{2l}{\tilde{\varpi }}^{2}+\frac{l}{2}\varpi^{*2}\right)\leq \frac{-\left(2l-1\right)}{2l}{\tilde{\varpi }}^{2}+\frac{l}{2}\varpi^{*2}. \end{array}$$

Furthermore, there exists a real number $\bar{k}>0$ to obtain

(16) $$\begin{array}{c} \bar{k}\tilde{\varpi }\hat{\varpi }+\frac{\bar{k}\left(2l-1\right)}{2l}{\tilde{\varpi }}^{2}\leq \frac{\bar{k}l}{2}\varpi^{*2}. \end{array}$$

### 2.3. Problem Formulation

**Figure 1:**
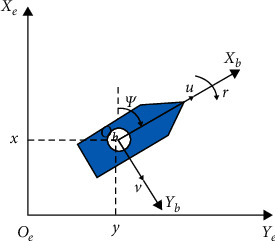
Motion of MSV.

Figure 1 depicts a plane model diagram of the MSV, which contains two commonly defined coordinate reference systems. The MSV model consisting of kinematics and dynamics is represented with reference to the following:

(17) $$\begin{array}{c} \left\{\begin{array}{c} \dot{n}=R\left(\psi \right)\nu \\ M\dot{\nu }+C\left(\nu \right)\nu +D\left(\nu \right)\nu =\tau +MR^{T}\left(\psi \right)d\left(t\right) \end{array}\right., \end{array}$$

where *n*=[*x*, *y*, *ψ*]^*T*^ represents the position, and (*x*, *y*) and *ψ* denote the position and yaw angle in the earth-fixed (EF) frame, respectively. The vector *ν*=[*u*, *v*, *r*]^*T*^ denotes the velocity in the surge, sway, and yaw directions in the body-fixed (BF) frame. *M* ∈ *R*^3×3^, *C*(*ν*) ∈ *R*^3×3^, and *D*(*ν*) ∈ *R*^3×3^ denote the mass matrix and the total Coriolis and centripetal acceleration matrix, respectively. *τ*=[*τ*_*u*_, *τ*_*v*_, *τ*_*r*_]^*T*^ is the control input of the MSV system. The vector *d*(*t*) represents external disturbance. *R*(*ψ*) represents the rotation matrix as follows:

(18) $$\begin{array}{c} R\left(\psi \right)=\left[\begin{array}{ccc} \cos\psi & -\sin\psi & 0\\ \sin\psi & \cos\psi & 0\\ 0 & 0 & 1 \end{array}\right], \end{array}$$

with the properties: *R*^*T*^(*ψ*)=*R*^−1^(*ψ*), $\dot{R}\left(\psi \right)=R\left(\psi \right)S\left(r\right)$, and

(19) $$\begin{array}{c} S\left(r\right)=\left[\begin{array}{ccc} 0 & -r & 0\\ r & 0 & 0\\ 0 & 0 & 0 \end{array}\right], \end{array}$$

where *S*^*T*^(*r*)=−*S*(*r*), *R*^*T*^(*ψ*)*S*(*r*)*R*(*ψ*)=*R*(*ψ*)*S*(*r*)*R*^*T*^(*ψ*)=*S*(*r*).

*Coordinate Transformation*. In the EF frame, the desired trajectory is defined as *x*_*d*_, *y*_*d*_, and *ψ*_*d*_ represents the desired heading angle. Therefore, the desired velocity *ν*_*d*_ can be obtained by kinematics as follows:

(20) $$\begin{array}{c} \nu_{d}=R^{T}\left(\psi_{d}\right){\dot{n}}_{d}, \end{array}$$

where the desired trajectory *n*_*d*_=[*x*_*d*_, *y*_*d*_, *ψ*_*d*_]^*T*^, *ν*_*d*_=[*u*_*d*_, *v*_*d*_, *r*_*d*_]^*T*^. According to *R*^*T*^(*ψ*_*d*_)=*R*^−1^(*ψ*_*d*_) and ${\dot{R}}^{T}\left(\psi_{d}\right)=S^{T}\left(r_{d}\right)R^{T}\left(\psi_{d}\right)$, computing the time derivative of (20) yields

(21) $$\begin{array}{c} {\dot{\nu }}_{d}={\dot{R}}^{T}\left(\psi_{d}\right){\dot{n}}_{d}+R^{T}\left(\psi_{d}\right){\ddot{n}}_{d}=R^{T}\left(\psi_{d}\right)\left({\ddot{n}}_{d}-R\left(\psi_{d}\right)S\left(r_{d}\right)\nu_{d}\right). \end{array}$$

Define *n*_*e*_ and *ν*_*e*_ as follows: *n*_*e*_=*n* − *n*_*d*_, *ν*_*e*_=*ν* − *ν*_*d*_. Then, system (17) transforms into the following:

(22) $$\begin{array}{c} \left\{\begin{array}{c} {\dot{n}}_{e}=\left[R\left(\psi \right)-R\left(\psi_{d}\right)\right]\nu_{d}+R\left(\psi \right)\nu_{e}\\ M{\dot{\nu }}_{e}=-C\left(\nu \right)\nu -D\left(\nu \right)\nu +\tau +R^{T}\left(\psi \right)d\left(t\right)-M{\dot{v}}_{d.} \end{array}\right.. \end{array}$$

Letting *x*_1_=*n*_*e*_ and $x_{2}={\dot{x}}_{1}={\dot{n}}_{e}$, (22) can be rewritten as

(23) $$\begin{array}{c} \left\{\begin{array}{c} {\dot{x}}_{1}=x_{2}\\ {\dot{x}}_{2}=f\left(x_{1},x_{2}\right)+R\left(\psi \right)M^{-1}\tau +d\left(t\right) \end{array}\right., \end{array}$$

with

(24) $$\begin{array}{c} f\left(x_{1},x_{2}\right)=\left[R\left(\psi \right)S\left(r\right)-R\left(\psi_{d}\right)S\left(r_{d}\right)\right]\nu_{d}+\left[R\left(\psi \right)-R\left(\psi_{d}\right)\right]{\dot{\nu }}_{d}\\ +S\left(r\right)x_{2}+R\left(\psi \right)M^{-1}\left[-C\left(\nu \right)\nu -D\left(\nu \right)\nu -M{\dot{\nu }}_{d}\right]-S\left(r\right)\left[R\left(\psi \right)-R\left(\psi_{d}\right)\right]\nu_{d}. \end{array}$$

## 3. Main Results

In this part, the stability of the fixed-time H_∞_ controller is analyzed, and the fixed-time stability combined with the H_∞_ control scheme is applied to the MSV system for the first time. Then, the SSNN is used to fit lumped disturbances consisting of environmental disturbances, AF and uncertainties. Finally, the design of the SSNN algorithm structure is introduced in detail.

### 3.1. Robust Fixed-Time H∞ Lyapunov Stability

**Theorem 1 .**

For 8, there is a positive definite function *V*(*x*) near the origin *U*^*∗*^ ⊂ *R*^*n*^ and real numbers *κ*_1_ > 0, *κ*_2_ > 0 and 0 < *α* < 1, *β* > 1, which satisfies

(25) $$\begin{array}{c} \dot{V}\left(x\right)+\kappa_{1}V^{\alpha }\left(x\right)+\kappa_{2}V^{\beta }\left(x\right)\leq \frac{1}{2}\left(\gamma^{2}{\left\|\omega \right\|}^{2}-{\left\|z\right\|}^{2}\right),\forall x\in \frac{U^{*}}{\left\{0\right\}}. \end{array}$$

Then, system (8) has an *L*_2_ gain of equal to or less than *γ*, and it is locally FTS at the origin. Assuming that *U*^*∗*^=*R*^*n*^ and *V*(*x*) are radially unbounded, then system (8) is globally FTS at the origin.

**Proof**

(i) When *ω*=0, (25) obtains the following result:
  (26) $$\begin{array}{c} \dot{V}\left(x\right)+\kappa_{1}V^{\alpha }\left(x\right)+\kappa_{2}V^{\beta }\left(x\right)\leq \frac{1}{2}\left(\gamma^{2}{\left\|\omega \right\|}^{2}-{\left\|z\right\|}^{2}\right)=-\frac{1}{2}{\left\|z\right\|}^{2}\leq 0. \end{array}$$
According to Lemma 1, system (8) is FTS.
(ii) When *ω* ≠ 0 and *V*(*x*) > 0,

(27) $$\begin{array}{c} \dot{V}\left(x\right)\leq \dot{V}\left(x\right)+\kappa_{1}V^{\alpha }\left(x\right)+\kappa_{2}V^{\beta }\left(x\right)\leq \frac{1}{2}\left(\gamma^{2}{\left\|\omega \right\|}^{2}-{\left\|z\right\|}^{2}\right). \end{array}$$

According to Definition 2, the *L*_2_ gain of the system is less than or equal to *γ*. The proof is complete.

### 3.2. Design of Fixed-Time H∞ Controller for MSVs

Analyze the error dynamic system (23), design auxiliary controllers *ϑ*_1_(*x*_1_) ∈ *R*^*n*^ and *ϑ*_1_(0)=0, and define error vector *z*_1_ as follows:

(28) $$\begin{array}{c} z_{1}=x_{2}-\vartheta_{1}\left(x_{1}\right). \end{array}$$

Substituting (28) into system (23), a new error dynamic system is obtained as

(29) $$\begin{array}{c} \left\{\begin{array}{c} {\dot{x}}_{1}=z_{1}+\vartheta_{1}\left(x_{1}\right),\\ {\dot{z}}_{1}=R\left(\psi \right)M^{-1}\tau +f\left(x_{1},x_{2}\right)+d\left(t\right)-{\dot{\vartheta }}_{1}\left(x_{1}\right). \end{array}\right.. \end{array}$$

To achieve fixed-time stability, the auxiliary controller functions are designed as

(30) $$\begin{array}{c} \left\{\begin{array}{c} \vartheta_{1}\left(x_{1}\right)=-p_{0}x_{1}-p_{1}Sig{\left(x_{1}\right)}^{\alpha }-p_{2}Sig{\left(x_{1}\right)}^{\beta }\\ \vartheta_{2}\left(z_{1}\right)=-p_{3}Sig{\left(z_{1}\right)}^{\alpha }-p_{4}Sig{\left(z_{1}\right)}^{\beta } \end{array}\right., \end{array}$$

where *p*_*i*_(*i*=0,1,2,3,4)=diag(*p*_*i*1_, *p*_*i*2_,…, *p*_*in*_) are positive 0 < *α* < 1, *β* > 1. For (29), a vector of the performance metric is defined as

(31) $$\begin{array}{c} z=\left[\begin{array}{c} \lambda_{1}x_{1}\\ \lambda_{2}z_{1} \end{array}\right], \end{array}$$

where *λ*_ 1_ > 0 and *λ*_ 2_ > 0 are the weighted coefficients of *x*_1_ and *z*_1_, respectively.

**Theorem 2 .**

**Figure 2:**
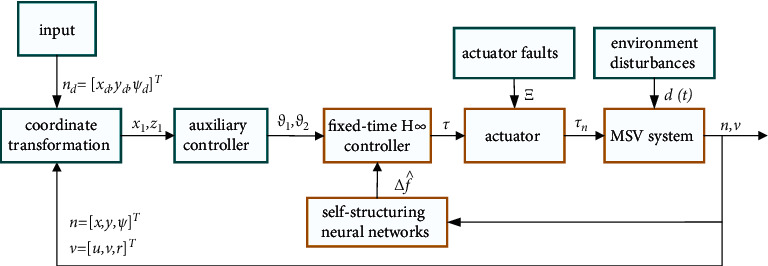
Schematic of fixed-time H_∞_ control system for MSV.

If all the assumptions are satisfied. For error systems (29), the following H_∞_ control law is designed as

(32) $$\begin{array}{c} \tau =MR{\left(\psi \right)}^{T}\left[{\dot{\vartheta }}_{1}\left(x_{1}\right)+\vartheta_{2}\left(z_{1}\right)-x_{1}-\left(\frac{1}{2\gamma^{2}}+\frac{\lambda_{2}^{2}}{2}\right)z_{1}-f\left(x_{1},x_{2}\right)\right], \end{array}$$

and we can choose the appropriate parameters so that the output of system (29) is FTS and satisfies *L*_2_ gain less than or equal to *γ*. The block diagram of the algorithm-based system is shown in Figure 2.

**Proof**

Substituting (32) into (29), we have

(33) $$\begin{array}{c} \left\{\begin{array}{c} {\dot{x}}_{1}=z_{1}+\vartheta_{1}\left(x_{1}\right),\\ {\dot{z}}_{1}=\vartheta_{2}\left(z_{1}\right)+d\left(t\right)-x_{1}-\left(\frac{1}{2\gamma^{2}}+\frac{\lambda_{2}^{2}}{2}\right)z_{1.} \end{array}\right.. \end{array}$$

**Step 1 .**

Construct a Lyapunov function of the form as

(34) $$\begin{array}{c} V_{1}=\frac{1}{2}x_{1}^{T}x_{1}+\frac{1}{2}z_{1}^{T}z_{1}. \end{array}$$

Substitute (33) into the derivative of (34). Then,

(35) $$\begin{array}{c} {\dot{V}}_{1}=x_{1}^{T}{\dot{x}}_{1}+z_{1}^{T}{\dot{z}}_{1}=x_{1}^{T}\vartheta_{1}\left(x_{1}\right)+z_{1}^{T}\vartheta_{2}\left(z_{1}\right)+z_{1}^{T}d\left(t\right)-z_{1}^{T}\left(\frac{1}{2\gamma^{2}}+\frac{\lambda_{2}^{2}}{2}\right)z_{1}. \end{array}$$

**Step 2 .**

The objective is to prove that the *L*_2_ gain of the H_∞_ control is equal to or less than *γ* by defining the function Η_1_ as follows:

(36) $$\begin{array}{c} {Η}_{1}={\dot{V}}_{1}+\frac{1}{2}\left({\left\|z\right\|}^{2}-\gamma^{2}{\left\|d\right\|}^{2}\right). \end{array}$$

Combining (33), (35), (36), and choice *p*^*∗*^ ≥ *λ*_1_^2^/2 and *p*^*∗*^=min{*p*_0*i*_}, then

(37) $$\begin{array}{c} {Η}_{1}=x_{1}^{T}\vartheta_{1}\left(x_{1}\right)+z_{1}^{T}\vartheta_{2}\left(z_{1}\right)-z_{1}^{T}\left(\frac{1}{2\gamma^{2}}+\frac{\lambda_{2}^{2}}{2}\right)z_{1}+z_{1}^{T}d+\frac{1}{2}\left({\left\|z\right\|}^{2}-\gamma^{2}{\left\|d\right\|}^{2}\right)\\ =-x_{1}^{T}p_{0}x_{1}-x_{1}^{T}p_{1}Sig{\left(x_{1}\right)}^{\alpha }-x_{1}^{T}p_{2}Sig{\left(x_{1}\right)}^{\beta }-z_{1}^{T}p_{3}Sig{\left(z_{1}\right)}^{\alpha }-z_{1}^{T}p_{4}Sig{\left(z_{1}\right)}^{\beta }+z_{1}^{T}d-z_{1}^{T}\frac{1}{2\gamma^{2}}z_{1}+\frac{1}{2}\lambda_{1}^{2}{\left\|x_{1}\right\|}^{2}-\frac{1}{2}\gamma^{2}{\left\|d\right\|}^{2}\\ \leq -x_{1}^{T}p_{0}x_{1}-x_{1}^{T}p_{1}Sig{\left(x_{1}\right)}^{\alpha }-x_{1}^{T}p_{2}Sig{\left(x_{1}\right)}^{\beta }-z_{1}^{T}p_{3}Sig{\left(z_{1}\right)}^{\alpha }-z_{1}^{T}p_{4}Sig{\left(z_{1}\right)}^{\beta }+\left\|z_{1}\right\|\left\|d\right\|-\frac{1}{2\gamma^{2}}{\left\|z_{1}\right\|}^{2}+\frac{1}{2}\lambda_{1}^{2}{\left\|x_{1}\right\|}^{2}-\frac{1}{2}\gamma^{2}{\left\|d\right\|}^{2}\\ =-x_{1}^{T}p_{0}x_{1}-x_{1}^{T}p_{1}Sig{\left(x_{1}\right)}^{\alpha }-x_{1}^{T}p_{2}Sig{\left(x_{1}\right)}^{\beta }-z_{1}^{T}p_{3}Sig{\left(z_{1}\right)}^{\alpha }-z_{1}^{T}p_{4}Sig{\left(z_{1}\right)}^{\beta }+\frac{1}{2}\lambda_{1}^{2}{\left\|x_{1}\right\|}^{2}-{\left(\frac{1}{\sqrt{2}\gamma }z_{1}-\frac{\gamma }{\sqrt{2}}d\right)}^{T}\left(\frac{1}{\sqrt{2}\gamma }z_{1}-\frac{\gamma }{\sqrt{2}}d\right)\\ \leq -x_{1}^{T}p_{1}Sig{\left(x_{1}\right)}^{\alpha }-x_{1}^{T}p_{2}Sig{\left(x_{1}\right)}^{\beta }-z_{1}^{T}p_{3}Sig{\left(z_{1}\right)}^{\alpha }-z_{1}^{T}p_{4}Sig{\left(z_{1}\right)}^{\beta }-\left(p^{*}-\frac{1}{2}\lambda_{1}^{2}\right){\left\|x_{1}\right\|}^{2}\\ \leq -x_{1}^{T}p_{1}Sig{\left(x_{1}\right)}^{\alpha }-z_{1}^{T}p_{3}Sig{\left(z_{1}\right)}^{\alpha }-x_{1}^{T}p_{2}Sig{\left(x_{1}\right)}^{\beta }-z_{1}^{T}p_{4}Sig{\left(z_{1}\right)}^{\beta }. \end{array}$$

Assuming that *σ*=(1+*α*)/2, 1/2 < *σ* < 1, *δ*=(1+*β*)/2, *δ* > 1, *p*_1min_=min{*p*_1*i*_}, *p*_2min_=min{*p*_2*i*_}, *p*_3min_=min{*p*_3*i*_}, *p*_4min_=min{*p*_4*i*_}, ${\tilde{p}}_{1}=2^{\sigma }p_{1\min}$, ${\tilde{p}}_{2}=2^{\delta }p_{2\min}$, ${\tilde{p}}_{3}=2^{\sigma }p_{3\min}$, ${\tilde{p}}_{4}=2^{\delta }p_{4\min}$, ${\bar{p}}_{\text{a}}=\min\left\{{\tilde{p}}_{1},{\tilde{p}}_{3}\right\}$, and ${\overset{⌢}{p}}_{\text{a}}=\min\left\{{\tilde{p}}_{2},{\tilde{p}}_{4}\right\}$ according to Lemma 3, inequality (37) can be reduced to

(38) $$\begin{array}{c} {Η}_{1}\leq -x_{1}^{T}p_{1}Sig{\left(x_{1}\right)}^{\alpha }-z_{1}^{T}p_{3}Sig{\left(z_{1}\right)}^{\alpha }-x_{1}^{T}p_{2}Sig{\left(x_{1}\right)}^{\beta }-z_{1}^{T}p_{4}Sig{\left(z_{1}\right)}^{\beta }\\ \leq -p_{1\min}\sum_{i=1}^{n}{\left|x_{1i}\right|}^{1+\alpha }-p_{3\min}\sum_{i=1}^{n}{\left|z_{1i}\right|}^{1+\alpha }-p_{2\min}\sum_{i=1}^{n}{\left|x_{1i}\right|}^{1+\beta }-p_{4\min}\sum_{i=1}^{n}{\left|z_{1i}\right|}^{1+\beta }\\ \leq -p_{1\min}\sum_{i=1}^{n}{\left|x_{1i}^{2}\right|}^{\left(1+\alpha \right)/2}-p_{3\min}\sum_{i=1}^{n}{\left|z_{1i}^{2}\right|}^{\left(1+\alpha \right)/2}-p_{2\min}\sum_{i=1}^{n}{\left|x_{1i}^{2}\right|}^{\left(1+\beta \right)/2}-p_{4\min}\sum_{i=1}^{n}{\left|z_{1i}^{2}\right|}^{\left(1+\beta \right)/2}.\\ \leq -{\tilde{p}}_{1}{\left(\frac{1}{2}\sum_{i=1}^{n}x_{1i}^{2}\right)}^{\sigma }-{\tilde{p}}_{3}{\left(\frac{1}{2}\sum_{i=1}^{n}z_{1i}^{2}\right)}^{\sigma }-{\tilde{p}}_{2}{\left(\frac{1}{2}\sum_{i=1}^{n}x_{1i}^{2}\right)}^{\delta }-{\tilde{p}}_{4}{\left(\frac{1}{2}\sum_{i=1}^{n}z_{1i}^{2}\right)}^{\delta }\\ \leq -{\bar{p}}_{a}V_{1}^{\delta }-{\overset{⌢}{p}}_{a}V_{1}^{\delta } \end{array}$$

Combining (36) and (38), the following conclusions can be obtained:

(39) $$\begin{array}{c} {\dot{V}}_{1}+{\bar{p}}_{\text{a}}V_{1}^{\sigma }+{\overset{⌢}{p}}_{\text{a}}V_{1}^{\delta }\leq \frac{1}{2}\left(\gamma^{2}{\left\|d\right\|}^{2}-{\left\|z\right\|}^{2}\right). \end{array}$$

From Theorem 1, error system (29) is FTS at the origin, and *V*_1_(*x*)⟶*∞* holds when *x*_1_⟶*∞*,  *z*_1_⟶*∞*. Thus, system (29) achieves FTS under the action of controller (32), while the *L*_2_ gain satisfies the constraint of being less than or equal to *γ*. The settling time satisfies

(40) $$\begin{array}{c} T_{1}\leq \frac{1}{{\bar{p}}_{\text{a}}\left(1-\sigma \right)}+\frac{1}{{\overset{⌢}{p}}_{\text{a}}\left(\delta -1\right)}. \end{array}$$

The proof is complete, and MSV system (17) has a fast response, strong robustness, and fixed-time stability under the action of controller (32).

**Remark 2 .**

In ${\dot{\vartheta }}_{1}\left(x_{1}\right)$, when *x*_1*i*_=0 and ${\dot{x}}_{1i}\neq 0$, the derivative of *Sig*(*x*_1_)^*α*^ is infinite, which leads to singularities in the system. To avoid this problem, a threshold Δ is introduced to judge the singularity, ${\dot{\vartheta }}_{1}\left(x_{1}\right)$ is redefined as follows, and ${\dot{\vartheta }}_{1}\left(x_{1}\right)$ is redefined as follows:

(41) $$\begin{array}{c} {\dot{\vartheta }}_{i}\left(x_{1i}\right)=\left\{\begin{array}{cc} -p_{1i}\alpha {\left|x_{1i}\right|}^{\alpha -1}{\dot{x}}_{1i}-p_{2i}\beta {\left|x_{1i}\right|}^{\beta -1}{\dot{x}}_{1i}, & \text{if}\left|x_{1i}\right|\geq \Delta \text{and}{\dot{x}}_{1i}\neq 0,\\ -p_{1i}\alpha {\left|{ℜ}_{i}\right|}^{\alpha -1}{\dot{x}}_{1i}-p_{2i}\beta {\left|{ℜ}_{i}\right|}^{\beta -1}{\dot{x}}_{1i}, & \text{if}\left|x_{1i}\right|<\Delta \text{and}{\dot{x}}_{1i}\neq 0,\\ 0, & \text{if}{\dot{x}}_{1i}=0, \end{array}\right. \end{array}$$

where Δ > 0 and *ℜ*_*i*_ > 0, and Δ takes a value in (0.7, 0.9).

**Remark 3 .**

If all assumptions are satisfied, for error systems (29), the composite robust controller is designed as (32), there exist design parameters *α*, *β*, *γ*, *λ*_ 1_, *λ*_ 2_ such that all outputs of error systems (29) are FTS, and the tracking error eventually converges to a small region around zero.

### 3.3. Self-Structuring Neural Network (SSNN) Control

Considering that the unknown Δ*C*(*v*), Δ*D*(*v*) of the vessel system are uncertain, AF usually appears in many MSVs. In combination with (4), system (29) can be written as

(42) $$\begin{array}{c} \left\{\begin{array}{c} {\dot{x}}_{1}=z_{1}+\vartheta_{1}\left(x_{1}\right),\\ {\dot{z}}_{1}=R\left(\psi \right)M^{-1}\tau +f_{0}\left(x_{1},x_{2}\right)+\Delta f-{\dot{\vartheta }}_{1}\left(x_{1}\right) \end{array}\right., \end{array}$$

with

(43) $$\begin{array}{c} f_{0}\left(x_{1},x_{2}\right)=\left[R\left(\psi \right)S\left(r\right)-R\left(\psi_{d}\right)S\left(r_{d}\right)\right]\nu_{d}+\left[R\left(\psi \right)-R\left(\psi_{d}\right)\right]{\dot{\nu }}_{d}+S\left(r\right)x_{2}\\ +R\left(\psi \right)M^{-1}\left[-C_{0}\left(\nu \right)\nu -D_{0}\left(\nu \right)\nu -M{\dot{\nu }}_{d}\right]-S\left(r\right)\left[R\left(\psi \right)-R\left(\psi_{d}\right)\right]\nu_{d}, \end{array}$$

and Δ*f*=−*R*(*ψ*)*M*^−1^[Δ*C*(*ν*)*ν*+Δ*D*(*ν*)*ν*]+Ξ+*d*(*t*), $\Xi =R\left(\psi \right)M^{-1}B\left(t-t_{0}\right)\left(\left(E-I\right)\tau +{\bar{\tau }}_{n}\right)$, *B*(*t* − *t*_0_)=diag(*b*_1_(*t* − *t*_01_), *b*_2_(*t* − *t*_02_), *b*_3_(*t* − *t*_03_)), *E*=diag(*e*_11_, *e*_22_, *e*_33_), ${\bar{\tau }}_{n}={\left[{\bar{\tau }}_{n1},{\bar{\tau }}_{n2},{\bar{\tau }}_{n3}\right]}^{T}$, and the actual control input *τ*_*n*_=*τ*+Ξ. Δ*f* is the lumped disturbances, including the parametric uncertainties, actuator faults, and environmental disturbances. To solve the problem of lumped disturbances Δ*f* influence on control system, the SSNN is designed to fit the unknown dynamic equation as follows:

(44) $$\begin{array}{c} \Delta f=W^{*T}\Phi \left(\chi \right)+\varepsilon \left(\chi \right), \end{array}$$

where *W*^*∗*^=[*W*_*u*_^*∗*^, *W*_*v*_^*∗*^, *W*_*r*_^*∗*^]^*T*^, $\hat{W}$ is defined as the estimated value of the weight, $\tilde{W}$ represents the error of the weight, and $\tilde{W}=W^{*}-\hat{W}$. $\left|\varepsilon_{i}\right|\leq {\bar{\varepsilon }}_{i}$, and $\bar{\varepsilon }={\left[{\bar{\varepsilon }}_{1},{\bar{\varepsilon }}_{2},{\bar{\varepsilon }}_{3}\right]}^{T}$ is the unknown constant vector.

Substituting (44) into (42), the closed-loop system is obtained as

(45) $$\begin{array}{c} \left\{\begin{array}{c} {\dot{x}}_{1}=z_{1}+\vartheta_{1}\left(x_{1}\right),\\ {\dot{z}}_{1}=R\left(\psi \right)M^{-1}\tau +f_{0}\left(x_{1},x_{2}\right)+W^{*T}\Phi \left(\chi \right)+\varepsilon \left(\chi \right)-{\dot{\vartheta }}_{1}\left(x_{1}\right). \end{array}\right.. \end{array}$$

**Theorem 3 .**

If all assumptions are satisfied, for error systems (45), the following H_∞_ control law is designed as

(46) $$\begin{array}{c} \tau =MR{\left(\psi \right)}^{T}\left[{\dot{\vartheta }}_{1}\left(x_{1}\right)+\vartheta_{2}\left(z_{1}\right)-x_{1}-\left(\frac{1}{2\gamma^{2}}+\frac{\lambda_{2}^{2}}{2}\right)z_{1}-f_{0}\left(x_{1},x_{2}\right)-{\hat{W}}^{T}\Phi \left(\chi \right)\right], \end{array}$$

where ${\hat{W}}^{T}\Phi \left(\chi \right)$ is an estimate of Δ*f*, and the estimate of Δ*f* is defined as $\Delta \hat{f}$. The weight update law is obtained as follows:

(47) $$\begin{array}{c} \dot{\hat{W}}=\Gamma \left(-\Phi z_{1}+\bar{k}\hat{W}\right), \end{array}$$

and we can choose the appropriate parameters so that the output of system (45) is PFTS and satisfies *L*_2_ gain less than or equal to *γ*.

**Proof**

Substituting (46) into (45), the closed-loop system is obtained as

(48) $$\begin{array}{c} \left\{\begin{array}{c} {\dot{x}}_{1}=z_{1}+\vartheta_{1}\left(x_{1}\right)\\ {\dot{z}}_{1}=\vartheta_{2}\left(z_{1}\right)-x_{1}-\left(\frac{1}{2\gamma^{2}}+\frac{\lambda_{2}^{2}}{2}\right)z_{1}+{\tilde{W}}^{T}\Phi \left(\chi \right)+\varepsilon . \end{array}\right.. \end{array}$$

A new Lyapunov function of the form is constructed as

(49) $$\begin{array}{c} V_{2}=\frac{1}{2}x_{1}^{T}x_{1}+\frac{1}{2}z_{1}^{T}z_{1}+\frac{1}{2}tr\left({\tilde{W}}^{T}\Gamma^{-1}\tilde{W}\right). \end{array}$$

Deriving *V*_2_ along with (47), we have

(50) $$\begin{array}{c} {\dot{V}}_{2}=x_{1}^{T}{\dot{x}}_{1}+z_{1}^{T}{\dot{z}}_{1}+tr\left({\tilde{W}}^{T}\Gamma^{-1}\dot{\hat{W}}\right)\\ =x_{1}^{T}\vartheta_{1}\left(x_{1}\right)+z_{1}^{T}\vartheta_{2}\left(z_{1}\right)-z_{1}^{T}\left(\frac{1}{2\gamma^{2}}+\frac{\lambda_{2}^{2}}{2}\right)z_{1}+z_{1}^{T}\varepsilon +z_{1}^{T}{\tilde{W}}^{T}\Phi +tr\left({\tilde{W}}^{T}\Gamma^{-1}\left(\Gamma \left(-\Phi z_{1}+\bar{k}\hat{W}\right)\right)\right)\\ =x_{1}^{T}\vartheta_{1}\left(x_{1}\right)+z_{1}^{T}\vartheta_{2}\left(z_{1}\right)-z_{1}^{T}\left(\frac{1}{2\gamma^{2}}+\frac{\lambda_{2}^{2}}{2}\right)z_{1}+z_{1}^{T}\varepsilon +\bar{k}tr\left({\tilde{W}}^{T}\hat{W}\right). \end{array}$$

A new function Η_2_ is established as

(51) $$\begin{array}{c} {Η}_{2}={\dot{V}}_{2}+\frac{1}{2}\left({\left\|z\right\|}^{2}-\gamma^{2}{\left\|\varepsilon \right\|}^{2}\right). \end{array}$$

Since ${\left\|\tilde{W}\right\|}_{F}$ is bounded, $\bar{k}\left(2l-1\right)/4l{\left\|\tilde{W}\right\|}_{F}^{2}\leq 1$ can be established by selecting appropriate parameters through analysis [34]. Combined with Lemma 4, we can obtain

(52) $$\begin{array}{c} {\left(\frac{\bar{k}\left(2l-1\right)}{4l}{\left\|\tilde{W}\right\|}_{F}^{2}\right)}^{\frac{\left(1+\alpha \right)}{2}}+\frac{\bar{k}{\left\|\tilde{W}\right\|}_{F}{\left\|\hat{W}\right\|}_{F}}{2}\leq \left(\frac{\bar{k}\left(2l-1\right)}{4l}{\left\|\tilde{W}\right\|}_{F}^{2}\right)+\frac{\bar{k}{\left\|\tilde{W}\right\|}_{F}{\left\|\hat{W}\right\|}_{F}}{2}\leq \frac{\bar{k}l}{4}{\left\|W^{*}\right\|}_{F}^{2}. \end{array}$$

Similarly,

(53) $$\begin{array}{c} {\left(\frac{\bar{k}\left(2l-1\right)}{4l}{\left\|\tilde{W}\right\|}_{F}^{2}\right)}^{\frac{\left(1+\beta \right)}{2}}+\frac{\bar{k}{\left\|\tilde{W}\right\|}_{F}{\left\|\hat{W}\right\|}_{F}}{2}\leq \frac{\bar{k}l}{4}{\left\|W^{*}\right\|}_{F}^{2}. \end{array}$$

Substituting (30), (48), (50), (52), and (53) into (51) and referring to the process in Section 3.2, we can obtain

(54) $$\begin{array}{c} {Η}_{2}=x_{1}^{T}\vartheta_{1}\left(x_{1}\right)+z_{1}^{T}\vartheta_{2}\left(z_{1}\right)-z_{1}^{T}\left(\frac{1}{2\gamma^{2}}+\frac{\lambda_{2}^{2}}{2}\right)z_{1}+z_{1}^{T}\varepsilon +\frac{1}{2}\left({\left\|z\right\|}^{2}-\gamma^{2}{\left\|\varepsilon \right\|}^{2}\right)+\bar{k}tr\left({\tilde{W}}^{T}\hat{W}\right)\\ =-x_{1}^{T}p_{0}x_{1}-x_{1}^{T}p_{1}Sig^{\alpha }\left(x_{1}\right)-x_{1}^{T}p_{2}Sig^{\beta }\left(x_{1}\right)-z_{1}^{T}p_{3}Sig^{\alpha }\left(z_{1}\right)-z_{1}^{T}p_{4}Sig^{\beta }\left(z_{1}\right)+z_{1}^{T}\varepsilon -z_{1}^{T}\frac{1}{2\gamma^{2}}z_{1}+\frac{1}{2}\lambda_{1}^{2}{\left\|x_{1}\right\|}^{2}-\frac{1}{2}\gamma^{2}{\left\|\varepsilon \right\|}^{2}\\ -{\left(\frac{\bar{k}\left(2l-1\right)}{4l}{\left\|\tilde{W}\right\|}_{F}^{2}\right)}^{\frac{\left(1+\alpha \right)}{2}}+{\left(\frac{\bar{k}\left(2l-1\right)}{4l}{\left\|\tilde{W}\right\|}_{F}^{2}\right)}^{\frac{\left(1+\alpha \right)}{2}}+\frac{\bar{k}{\left\|\tilde{W}\right\|}_{F}{\left\|\hat{W}\right\|}_{F}}{2}-{\left(\frac{\bar{k}\left(2l-1\right)}{4l}{\left\|\tilde{W}\right\|}_{F}^{2}\right)}^{\frac{\left(1+\beta \right)}{2}}+{\left(\frac{\bar{k}\left(2l-1\right)}{4l}{\left\|\tilde{W}\right\|}_{F}^{2}\right)}^{\frac{\left(1+\beta \right)}{2}}+\frac{\bar{k}{\left\|\tilde{W}\right\|}_{F}{\left\|\hat{W}\right\|}_{F}}{2}\\ \leq -x_{1}^{T}p_{0}x_{1}-x_{1}^{T}p_{1}Sig^{\alpha }\left(x_{1}\right)-x_{1}^{T}p_{2}Sig^{\beta }\left(x_{1}\right)-z_{1}^{T}p_{3}Sig^{\alpha }\left(z_{1}\right)-z_{1}^{T}p_{4}Sig^{\beta }\left(z_{1}\right)+\left\|z_{1}\right\|\left\|\varepsilon \right\|-\frac{1}{2\gamma^{2}}{\left\|z_{1}\right\|}^{2}\\ +\frac{1}{2}\lambda_{1}^{2}{\left\|x_{1}\right\|}^{2}-\frac{1}{2}\gamma^{2}{\left\|\varepsilon \right\|}^{2}-{\left(\frac{\bar{k}\left(2l-1\right)}{4l}{\left\|\tilde{W}\right\|}_{F}^{2}\right)}^{\frac{\left(1+\alpha \right)}{2}}+\frac{\bar{k}l}{4}{\left\|W^{*}\right\|}_{F}^{2}-{\left(\frac{\bar{k}\left(2l-1\right)}{4l}{\left\|\tilde{W}\right\|}_{F}^{2}\right)}^{\frac{\left(1+\beta \right)}{2}}+\frac{\bar{k}l}{4}{\left\|W^{*}\right\|}_{F}^{2}\\ \leq -x_{1}^{T}p_{0}x_{1}-x_{1}^{T}p_{1}Sig^{\alpha }\left(x_{1}\right)-x_{1}^{T}p_{2}Sig^{\beta }\left(x_{1}\right)-z_{1}^{T}p_{3}Sig^{\alpha }\left(z_{1}\right)-z_{1}^{T}p_{4}Sig^{\beta }\left(z_{1}\right)\\ +\frac{1}{2}\lambda_{1}^{2}{\left\|x_{1}\right\|}^{2}-{\left(\frac{1}{\sqrt{2}\gamma }z_{1}-\frac{\gamma }{\sqrt{2}}\varepsilon \right)}^{T}\left(\frac{1}{\sqrt{2}\gamma }z_{1}-\frac{\gamma }{\sqrt{2}}\varepsilon \right)-{\left(\frac{\bar{k}\left(2l-1\right)}{4l}{\left\|\tilde{W}\right\|}_{F}^{2}\right)}^{\frac{\left(1+\alpha \right)}{2}}-{\left(\frac{\bar{k}\left(2l-1\right)}{4l}{\left\|\tilde{W}\right\|}_{F}^{2}\right)}^{\frac{\left(1+\beta \right)}{2}}+\frac{\bar{k}l}{2}{\left\|W^{*}\right\|}_{F}^{2}\\ \leq -x_{1}^{T}p_{1}Sig^{\alpha }\left(x_{1}\right)-z_{1}^{T}p_{3}Sig^{\alpha }\left(z_{1}\right)-x_{1}^{T}p_{2}Sig^{\beta }\left(x_{1}\right)-z_{1}^{T}p_{4}Sig^{\beta }\left(z_{1}\right)-\left(p_{0}-\frac{1}{2}\lambda_{1}^{2}\right){\left\|x_{1}\right\|}^{2}\\ -{\left(\frac{\bar{k}\left(2l-1\right)}{4l}{\left\|\tilde{W}\right\|}_{F}^{2}\right)}^{\frac{\left(1+\alpha \right)}{2}}-{\left(\frac{\bar{k}\left(2l-1\right)}{4l}{\left\|\tilde{W}\right\|}_{F}^{2}\right)}^{\frac{\left(1+\beta \right)}{2}}+\frac{\bar{k}l}{2}{\left\|W^{*}\right\|}_{F}^{2}\\ \leq -x_{1}^{T}p_{1}Sig^{\alpha }\left(x_{1}\right)-z_{1}^{T}p_{3}Sig^{\alpha }\left(z_{1}\right)-x_{1}^{T}p_{2}Sig^{\beta }\left(x_{1}\right)-z_{1}^{T}p_{4}Sig^{\beta }\left(z_{1}\right)-{\left(\frac{\bar{k}\left(2l-1\right)}{4l}{\left\|\tilde{W}\right\|}_{F}^{2}\right)}^{\frac{\left(1+\alpha \right)}{2}}-{\left(\frac{\bar{k}\left(2l-1\right)}{4l}{\left\|\tilde{W}\right\|}_{F}^{2}\right)}^{\frac{\left(1+\beta \right)}{2}}+\frac{\bar{k}l}{2}{\left\|W^{*}\right\|}_{F}^{2}\\ \leq -p_{1\min}\sum_{i=1}^{n}{\left|x_{1i}\right|}^{1+\alpha }-p_{3\min}\sum_{i=1}^{n}{\left|z_{1i}\right|}^{1+\alpha }-p_{2\min}\sum_{i=1}^{n}{\left|x_{1i}\right|}^{1+\beta }-p_{4\min}\sum_{i=1}^{n}{\left|z_{1i}\right|}^{1+\beta }-{\left(\frac{\bar{k}\left(2l-1\right)}{4l}{\left\|\tilde{W}\right\|}_{F}^{2}\right)}^{\frac{\left(1+\alpha \right)}{2}}-{\left(\frac{\bar{k}\left(2l-1\right)}{4l}{\left\|\tilde{W}\right\|}_{F}^{2}\right)}^{\frac{\left(1+\beta \right)}{2}}+\frac{\bar{k}l}{2}{\left\|W^{*}\right\|}_{F}^{2}\\ \leq -p_{1\min}\sum_{i=1}^{n}{\left|x_{1i}^{2}\right|}^{\frac{\left(1+\alpha \right)}{2}}-p_{3\min}\sum_{i=1}^{n}{\left|z_{1i}^{2}\right|}^{\frac{\left(1+\beta \right)}{2}}-{\left(\frac{k\left(2l-1\right)}{4l}{\left\|\tilde{W}\right\|}_{F}^{2}\right)}^{\frac{\left(1+\alpha \right)}{2}}-p_{2\min}\sum_{i=1}^{n}{\left|x_{1i}^{2}\right|}^{\frac{\left(1+\beta \right)}{2}}\\ -p_{4\min}\sum_{i=1}^{n}{\left|z_{1i}^{2}\right|}^{\frac{\left(1+\beta \right)}{2}}-{\left(\frac{\bar{k}\left(2l-1\right)}{4l}{\left\|\tilde{W}\right\|}_{F}^{2}\right)}^{\frac{\left(1+\beta \right)}{2}}+\frac{\bar{k}l}{2}{\left\|W^{*}\right\|}_{F}^{2}\\ \leq -{\tilde{p}}_{1}{\left(\frac{1}{2}\sum_{i=1}^{n}x_{1i}^{2}\right)}^{\sigma }-{\tilde{p}}_{3}{\left(\frac{1}{2}\sum_{i=1}^{n}z_{1i}^{2}\right)}^{\sigma }-{\left(\frac{\bar{k}\left(2l-1\right)}{4l}{\left\|\tilde{W}\right\|}_{F}^{2}\right)}^{\sigma }-{\tilde{p}}_{2}{\left(\frac{1}{2}\sum_{i=1}^{n}x_{1i}^{2}\right)}^{\delta }-{\tilde{p}}_{4}{\left(\frac{1}{2}\sum_{i=1}^{n}z_{1i}^{2}\right)}^{\delta }-{\left(\frac{\bar{k}\left(2l-1\right)}{4l}{\left\|\tilde{W}\right\|}_{F}^{2}\right)}^{\delta }+\frac{\bar{k}l}{2}{\left\|W^{*}\right\|}_{F}^{2}\leq -{\bar{p}}_{\text{b}}V_{2}^{\sigma }-{\overset{⌢}{p}}_{\text{b}}V_{2}^{\delta }+\Pi , \end{array}$$

where

(55) $$\begin{array}{c} \left\{\begin{array}{c} {\bar{p}}_{b}=\min\left\{{\tilde{p}}_{1},{\tilde{p}}_{3},{\left(\frac{\bar{k}\left(2l-1\right)}{2l}\right)}^{\sigma }\right\},\\ {\overset{⌢}{p}}_{b}=\min\left\{{\tilde{p}}_{2},{\tilde{p}}_{4},{\left(\frac{\bar{k}\left(2l-1\right)}{2l}\right)}^{\delta }\right\},\\ \Pi =\frac{\bar{k}l}{2}{\left\|W^{*}\right\|}_{F}^{2}. \end{array}\right.. \end{array}$$

Combining (51) and (54), the following conclusions can be obtained:

(56) $$\begin{array}{c} {\dot{V}}_{2}+{\bar{p}}_{\text{b}}V_{2}^{\sigma }+{\overset{⌢}{p}}_{\text{b}}V_{2}^{\delta }\leq \frac{1}{2}\left(\gamma^{2}{\left\|\varepsilon \right\|}^{2}-{\left\|z\right\|}^{2}\right)+\Pi . \end{array}$$

The proof is complete.

From Lemma 2, error system (45) is PFTS at the origin, and *V*_1_(*x*)⟶*∞* holds when *x*_1_⟶*∞*,  *z*_1_⟶*∞*. Thus, system (45) achieves PFTS under the action of controller (46), while the *L*_2_ gain satisfies the constraint of being less than or equal to *γ*, and the error convergence range is established as follows:

(57) $$\begin{array}{c} x\in \left\{V\left(x\right)\leq \min\left\{{\left(\frac{1/2\left(\gamma^{2}{\left\|\varepsilon \right\|}^{2}-{\left\|z\right\|}^{2}\right)+\Pi }{\left(1-g\right)\kappa_{1}}\right)}^{1/\sigma },{\left(\frac{1/2\left(\gamma^{2}{\left\|\varepsilon \right\|}^{2}-{\left\|z\right\|}^{2}\right)+\Pi }{\left(1-g\right)\kappa_{2}}\right)}^{1/\delta }\right\}\right\}, \end{array}$$

and settling time is bounded by $T_{2}\leq 1/{\bar{p}}_{b}g\left(1-\sigma \right)+1/{\overset{⌢}{p}}_{b}g\left(\delta -1\right)$, where 0 < *g* < 1.

### 3.4. Parameter-Learning Algorithm of SSNN

**Figure 3:**
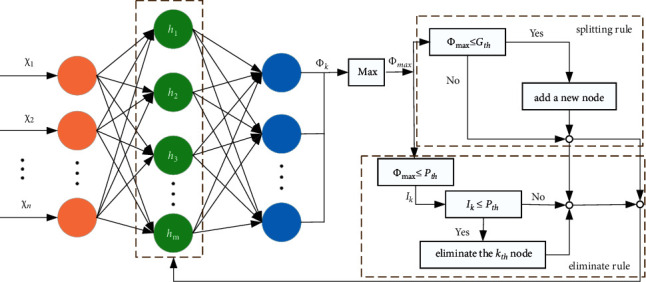
Flowchart of the self-structuring algorithm.

It is well-known that the fitting ability of a neural network is closely related to the network structure, and usually, it is difficult to determine the best network structure matched by the approximated nonlinear function. On the other hand, a change in the nonlinear function can also lead to a change in the optimal network structure. Therefore, this paper proposes an SSNN for solving this problem that can optimize the network structure online by splitting rules and censoring rules to achieve the best approximation effect while avoiding causing computational burden. The algorithm flow is shown in Figure 3:

(i) When the input signal of the neural network is far from the current neuron center, the network approximation ability is poor. At this time, it is necessary to add new nodes through the splitting rule to ensure the effective use of the signal and improve the fitting effect. The activation function value of the neural network is used to determine whether the neurons need to be split as follows:
  (58) $$\begin{array}{c} \Phi_{\max}=\underset{1\leq k\leq m}{\max}\Phi_{k},k=1,2,\ldots m. \end{array}$$
If Φ_max_ ≤ *G*_*th*_ is satisfied, then the neuron needs to be split, where *G*_*th*_ represents the split threshold. The parameters of the new neuron are as follows:
  (59) $$\begin{array}{c} \left\{\begin{array}{c} c_{k}^{\text{new}}=\frac{x+c_{k}^{\text{new}}}{2}\\ j_{k}^{\text{new}}=j_{k}\\ W_{k}^{\text{new}}=0 \end{array}\right., \end{array}$$
where *j*_*k*_ is a prespecified constant.
(ii) When a neural network fits a nonlinear function that is not complex, there are usually some neurons that are not effective for the approximation, and these unnecessary neurons should be removed to reduce the computational burden. The rules are as follows:

(60) $$\begin{array}{c} I_{k}=\left\{\begin{array}{cc} sI_{k}^{p}, & \text{if}\Phi_{k}<P_{tb}\\ 1, & \text{if}\Phi_{k}\geq P_{tb} \end{array}\right.k=1,2,\ldots ,m, \end{array}$$

where *s* is the decayed constant, *I*_*k*_ is the reference index, and *I*_*k*_^*p*^ denotes the most recent *I*_*k*_. *P*_*th*_ denotes the pregiven threshold; if *I*_*k*_ ≤ *P*_*th*_ holds, then the *k*th neuron is eliminated.

**Remark 4 .**

The splitting and eliminate thresholds are chosen reasonably by judging the complexity of the nonlinear function. If the nonlinear function is very complex, then a larger *G*_*th*_ value is selected to split more neurons to achieve a satisfactory approximation. If the nonlinear function is relatively simple, then a larger *P*_*th*_ value can be selected to delete more neurons, and the calculation amount can be reduced on the premise that the approximation effect meets the requirements.

## 4. Simulation Results

Through two comparative experiments, the influence of actuator faults on the controller is analyzed, and the superiority of the SSNN is verified.

### 4.1. Exploring Impact of AF on Control System

The influence of the AF on the control system is analyzed by observing the simulation results of the following three cases.

  Case (1): in designed controller (46), the part of SSNN fitting includes system uncertainties, external disturbances, and AF.
  Case (2): there are actuator faults in the system, but no processing is performed; that is, the SSNN only compensates for the uncertainty and disturbance.
  Case (3): there are no actuator faults in the system, the SSNN compensates for the uncertainty and disturbances, and the control rate is the same as Case (2).

The parameters of the ship are given in Table 1, and the disturbances refer to [35] as follows:

(61) $$\begin{array}{c} d\left(t\right)=\left[\begin{array}{c} 0.08\sin\left(0.1\pi t-\frac{\pi }{5}\right)\\ 0.1\sin\left(0.3\pi t+\frac{\pi }{6}\right)\\ 0.12\sin\left(0.2\pi t+\frac{\pi }{3}\right) \end{array}\right]. \end{array}$$

The desired trajectory is

(62) $$\begin{array}{c} \left\{\begin{array}{c} x_{d}=10\text{sin}\left(\text{0.}01\pi \text{t}+\frac{\pi }{360}\right)\\ y_{d}=8\text{sin}\left(\text{0.}02\pi \text{t}\right)\\ \psi_{d}=\arctan2\left({\dot{y}}_{d},{\dot{x}}_{d}\right) \end{array}\right.. \end{array}$$

This uncertainty can be expressed as Δ*C*=0.2*C*_0_, Δ*D*=0.2*D*_0_. The parameters for AF are given as *E*=diag(0.5, 0.5, 0.5), $\bar{\tau }={\left[10\text{,}\;10\text{,}\;4\right]}^{T}$, *t*_0_=[10,  15,  20]^*T*^, and *a*=[20,  10,  5]^*T*^. For other parameters, refer to Table 2.

**Figure 4:**
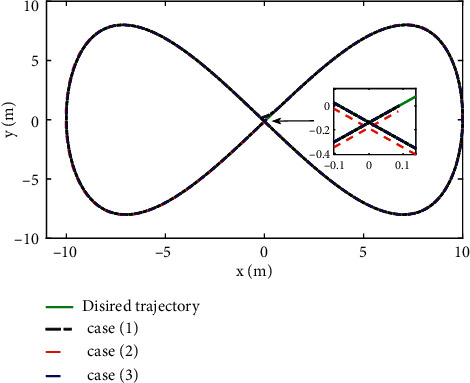
Actual and desired trajectories in EF frame.

**Figure 5:**
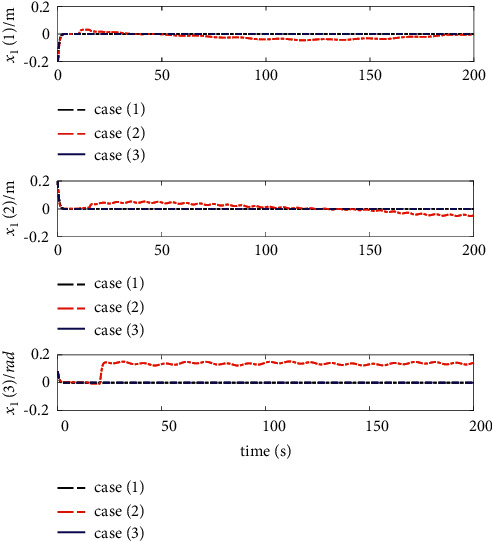
Position errors of trajectory tracking.

**Figure 6:**
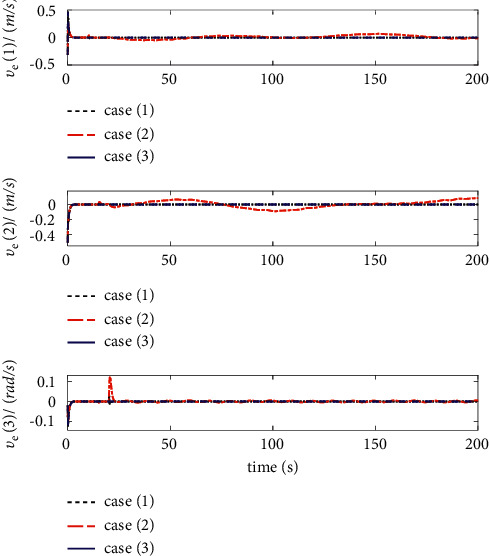
Velocity errors of trajectory tracking.

**Figure 7:**
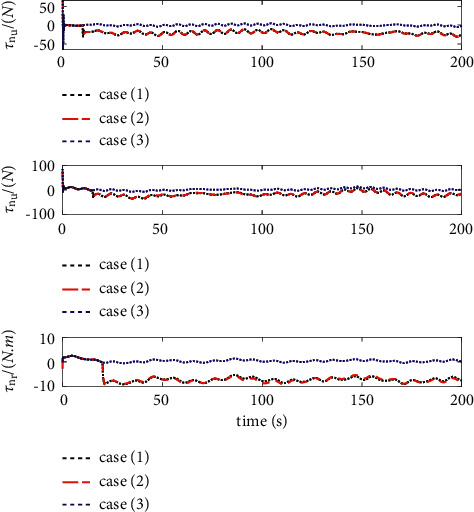
Control input.

**Figure 8:**
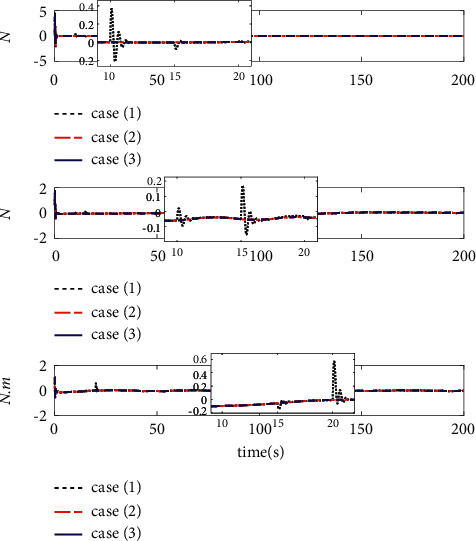
Estimated errors of system lumped disturbances Δ*f* by SSNN.

**Figure 9:**
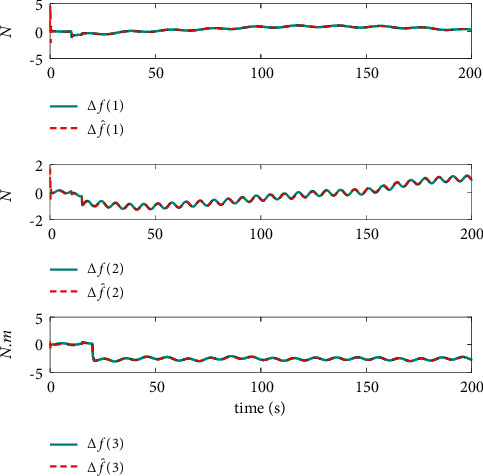
Fitting effect of SSNN in Case (1).

**Figure 10:**
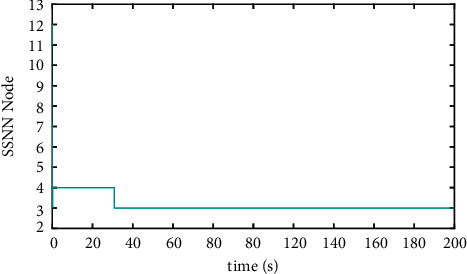
Number of SSNN nodes in Case (1).

**Figure 11:**
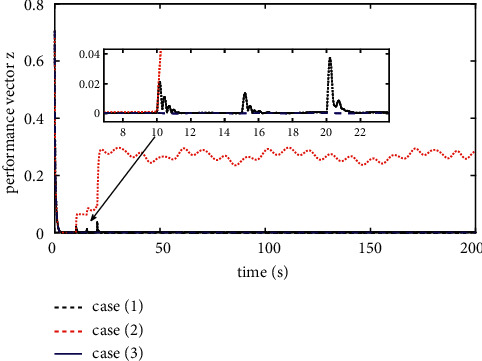
Norm of performance vector *z*.

The comparison results of the three cases are shown in Figure 4–11. Figure 4 shows the trajectory tracking effect of the MSV on the horizontal plane in three cases, and it can be found from the partial magnification that the tracking error is large due to the effect of AF in Case (2), while the effect of AF is well solved in the proposed controller in this paper, and the performance tracking effect is shown in Case (1). A more intuitive tracking error is shown in Figure 5. Combined with Cases (2) and (3), it can be judged that when actuator faults occur, the tracking performance of the controller will be greatly reduced.  Figure 6 shows the variation in the magnitude of the velocity tracking error. From Case (2), it can be found that the AF can also cause the tracking velocity to be difficult to maintain stable and always have some error with the desired velocity. The control inputs are shown in Figure 7, which perform within the engineering acceptable range and are stable and bounded.  Figure 8 shows the fitting errors of the SSNN.  Figure 9 shows the effect of the SSNN approaching lumped disturbances in Case (1).  Figure 10 shows the changes in the nodes of the network structure under the action of the SSNN algorithm, from the initial 11 nodes and finally stabilized to 3 nodes.  Figure 11 shows the curvature change of the performance metrics *z* of the proposed controller. It can be seen that *z* can still have fast convergence and strong robustness under the action of lumped disturbances.

### 4.2. Analyzing SSNN

**Figure 12:**
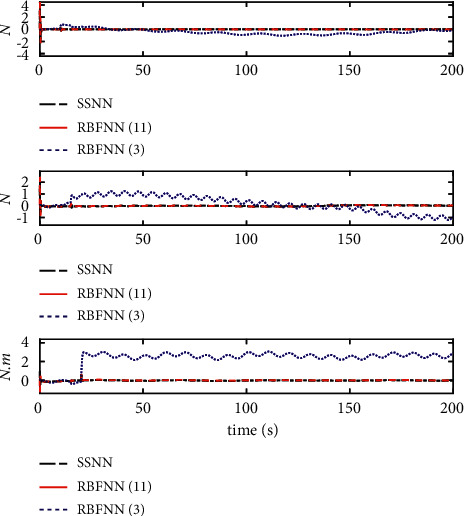
NN approximation error comparison.

**Figure 13:**
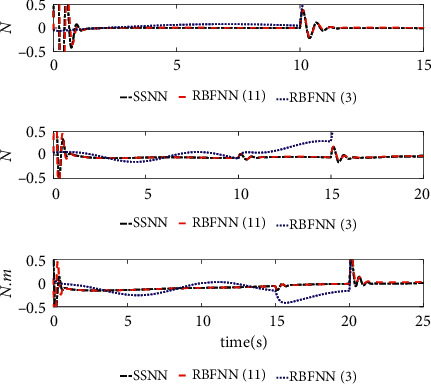
Detailed comparison of NN approximation errors.

**Figure 14:**
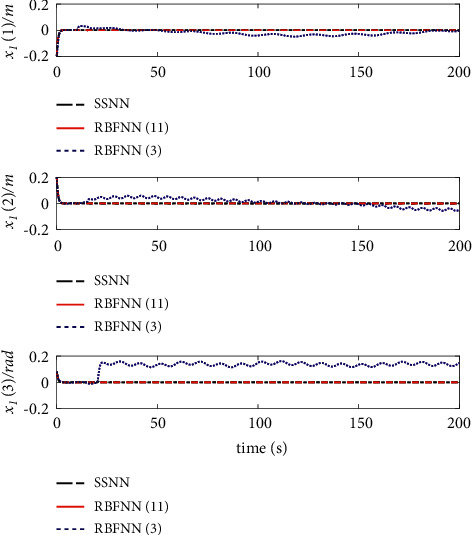
Position errors of trajectory tracking.

**Figure 15:**
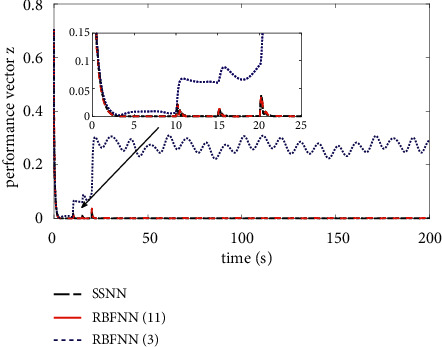
Norm of performance vector *z*.

**Figure 16:**
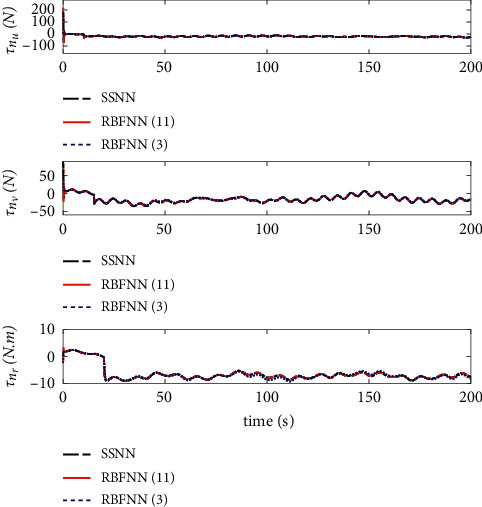
Control inputs.

From Figure 10, it can be seen that the initial value and stable value of the number of nodes of SSNN are 11 and 3, respectively. Therefore, the superiority of the SSNN is verified by comparing the fixed structure RBFNN with 11 nodes and 3 nodes. Figures 12–16 show a comparison of simulation results under the same experimental environment. Figures 12 and 13 show a comparison of NN fitting errors. In Figure 13, we find that due to the large fitting error at the beginning, when AF occurs, the estimation deviation of the RBFNN with only 3 nodes on the lumped disturbances becomes very large. In contrast, the SSNN control scheme showed good approximation ability. The tracking error of the controller also differs under the action of three different network structures of NNs, as shown in Figure 14. When AF occurs, both RBFNN and SSNN containing 11 nodes have good fitting ability, but RBFNN with only 3 nodes performs poorly.  Figure 15 shows the norm variation of the performance metric *z*. It can be seen that different network structures have a large impact on the performance of the controller, where the SSNN is able to reduce the computational burden while ensuring that the control performance is not affected.  Figure 16 clarifies that the corresponding control inputs under three different network structures are almost the same and meet the actual engineering requirements.

### 4.3. Scheme Comparison

To more intuitively and clearly reflect the advantages of the proposed control scheme, the trajectory tracking control method studied in this paper is defined as Strategy (1). The two typical control strategies are compared as follows:

  Strategy (2): finite-time stable strategy [31], the control law is designed by combining the TA-BLF and adaptive NN method, and its core parameters are *k*_1_=5, *k*_10_=1, *k*_11_=0.5 , *k*_2_=200, *k*_20_=100, and *k*_3_=10.
  Strategy (3): asymptotically stable strategy [30], the control law is designed by combining the asymmetric saturation actuators, adaptive NN and backstepping. Its core parameters are *k*_1_=diag{0.5, 0.5, 0.5}, *k*_2_=diag{300,300,300}, *k*_*w*_=0.1 and *k*_*ξ*_=0.5.

**Figure 17:**
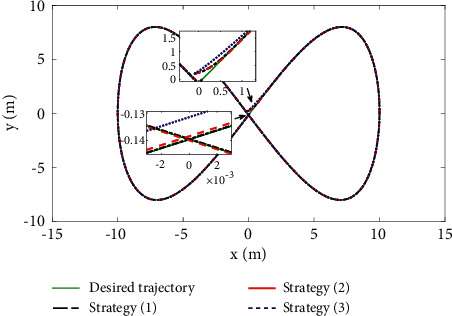
Trajectory tracking comparison.

**Figure 18:**
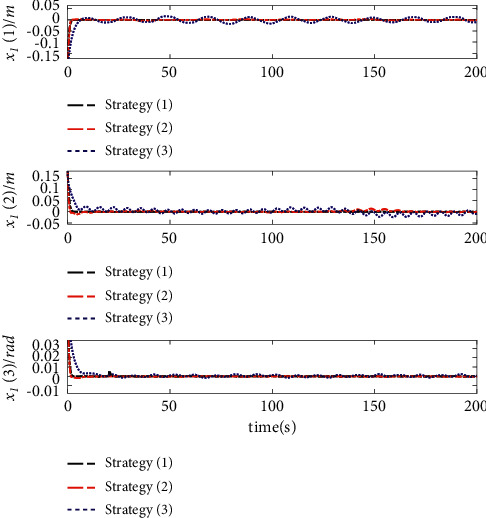
Tracking error comparison.

**Figure 19:**
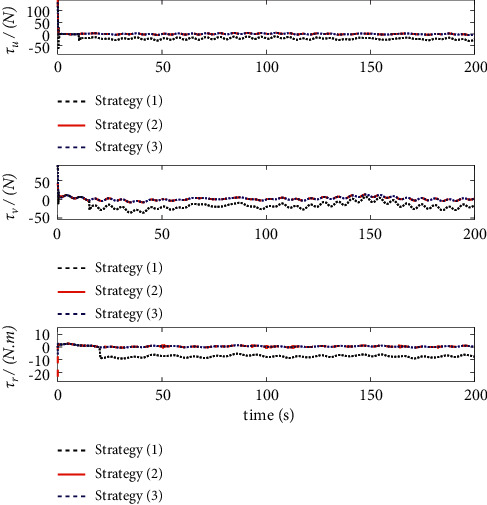
Control input comparison.

Note that this paper not only has the same model uncertainty and external disturbance but also considers the influence of actuator faults on the control system. A comparison of the simulation results of the three control strategies under the same initial value is shown in Figures 17-19.

The effect comparison of MSV autonomous tracking of the desired trajectory under three different control strategies is shown in Figure 17. The results clearly show that the control law designed in this paper has a faster response speed and higher tracking accuracy than Strategy (3).  Figure 18 shows a comparison of the tracking errors of the position and yaw angles under different strategies. Compared with Strategy (2) and Strategy (3), Control Strategy (1) proposed in this paper shows a relatively smooth tracking error curve, which indicates that the controller has stronger robustness, and the tracking error is also the smallest.  Figure 19 shows that the control inputs required by the three control strategies are almost the same before actuator failure. However, since the function of actuator faults is considered in this paper, it is necessary to improve the input of the system in the case of actuator faults to ensure the stability of the system. The results show that the control inputs required by the three strategies are implemented within the acceptable range of the project and are stable.

## 5. Conclusions

An adaptive robust fixed-time H_∞_ controller was proposed for the trajectory tracking control of MSV systems with unknown environmental disturbances, uncertainties, and actuator faults. The controller has an *L*_2_ gain, which presents stronger robustness and fast convergence. The SSNN not only has a good ability to approach unknown items but can also adjust the network structure by splitting or deleting nodes to save network resources. The effectiveness and superiority of the proposed controller were verified by comparing the results of simulation experiments. In future work, we will consider research on the robust control of MSV formations.

## Figures and Tables

**Table 1:** Vessel model parameters commonly used in research.

Parameters	Value	Parameters	Value
*M*_11_	25.8	*D*_11_	0.72+1.33|*u*|+5.87*u*^2^
*M*_22_	33.8	*D*_22_	0.8896+36.5|*v*|+0.805|*r*|
*M*_23_=*M*_32_	1.0115	*D*_23_	7.25+0.845|*v*|+3.45|*r*|
*M*_33_	2.76	*D*_32_	0.0313+3.96|*v*|+0.13|*r*|
*C*_13_=−*C*_31_	−33.8*v* − 1.0115*r*	*D*_33_	1.9 − 0.08|*v*|+0.75|*r*|
*C*_23_=−*C*_32_	25.8*u*		

**Table 2:** Parameters of controller and SSNN.

Controller parameters	Value	SSNN parameters	Value
*λ*_1_, *λ*_2_, *γ*	1	Γ	diag{150,150,150}
*α*	0.8	*k*	diag{0.01, 0.01, 0.01}
*β*	1.2	*G*_*th*_	0.8
*n*_*d*0_(initial)	[−0.1, 0.2, pi/2−0.5]^*T*^	*I*_*k*_(initial)	1
*v*_*d*0_(initial)	[0, 0, 0]^*T*^	*s*	0.5
*p*_0_, *p*_1_, *p*_2_	diag{0.5, 0.5, 0.5}	*P*_*th*_	0.2
*p*_3_, *p*_4_	diag{5,5,5}		

## Data Availability

The data used to support the findings of this study are included within the article.
